# Adult Manifestation of Milder Forms of Autism Spectrum Disorder; Autistic and Non-autistic Psychopathology

**DOI:** 10.1007/s10803-020-04403-9

**Published:** 2020-02-12

**Authors:** E. H. Horwitz, R. A. Schoevers, K. Greaves-Lord, A. de Bildt, C. A. Hartman

**Affiliations:** 1 University Medical Center Groningen, University of Groningen, Groningen, The Netherlands; 2 Lentis Mental Health, Groningen, The Netherlands; 3 University Medical Center Groningen, University Psychiatric Center, Hanzeplein 1, 9713 GZ Groningen, The Netherlands; 4 Present Address: GGZ Friesland, Leeuwarden, The Netherlands

**Keywords:** Autism spectrum disorder, Outcome, Young adult, Psychiatric comorbidity

## Abstract

We compared the presence of autistic and comorbid psychopathology and functional impairments in young adults who received a clinical diagnosis of Pervasive Developmental Disorders Not Otherwise Specified or Asperger’s Disorder during childhood to that of a referred comparison group. While the Autism Spectrum Disorder group on average scored higher on a dimensional ASD self- and other-report measure than clinical controls, the majority did not exceed the ASD cutoff according to the Autism Diagnostic Observation Schedule. Part of the individuals with an ASD diagnosis in their youth no longer show behaviors that underscribe a clinical ASD diagnosis in adulthood, but have subtle difficulties in social functioning and a vulnerability for a range of other psychiatric disorders.

## Introduction

There has been a sharp rise in the measured prevalence of autism spectrum disorder (ASD) in the last decade. This rise has been accompanied by an increased interest and investment in research on ASD. Yet, most studies were focused on children and research on ASD in young adulthood is still scarce (Hartman et al. 2016; Howlin and Magiati 2017). Limited data are available that describe topics like the symptom presentation of ASD in adulthood, the validity of diagnostic instruments in adulthood, the contribution of various sources of information to the diagnostic process (e.g. self- and other-report questionnaires, structured clinical observation), and comorbidity with other psychiatric disorders. Because these are important components of the (differential) diagnosis of autism in adulthood, achieving a better understanding of these topics is especially relevant for more accurate diagnostic assessment in clinical practice. This is the case in particular when we have to distinguish milder forms of ASD from other forms of psychopathology or abnormal behavior (Hartman et al. 2016). In the recent past, patients with milder forms of ASD often received a diagnosis of PDD-NOS or, to a much lesser extent, Asperger’s Disorder [according to the Diagnostic and Statistical Manual of Mental Disorders, fourth edition (DSM-IV, APA 2000)] in clinical care. The few available studies on the follow-up of these patients into adulthood suggest relatively low reliability and stability of the PDD-NOS classification (e.g. Luteijn et al. 2000; Rondeau et al. 2011) and significant overlap with features of other disorders such as Attention Deficit Hyperactivity Disorder (ADHD, Louwerse et al. 2015; Verheij et al. 2015). Studies on adult outcome for children with Asperger’s Disorder also are scarce and mainly showed a high rate of psychiatric comorbidity in this group (Gillberg et al. 2016). In the current paper we aim to explore the manifestation of ASD and comorbidity in adults who had a clinical diagnosis of milder ASD during childhood or adolescence according to multiple perspectives (i.e. structured observation, other- and self-report).

Prospective studies from childhood into adulthood are an important source of information on adult ASD. These have shown that the vast majority of individuals diagnosed with autism in early childhood still meet clinical criteria for autism in adulthood (e.g. McGovern and Sigman 2005; Billstedt et al. 2007). Seltzer et al. (2004) reviewed the retrospective, cross-sectional and prospective studies on symptom presentation and concluded that modest degrees of symptom abatement on all domains in adolescence and adulthood were the dominant pattern, but also found considerable variability in course patterns. Since that review, more longitudinal studies were published in which individuals with a diagnosis of autism in early childhood were followed into adulthood (Billstedt et al. 2007; Howlin et al. 2013; Anderson et al. 2014) with findings largely in line with the conclusions from the Seltzer review. Various symptoms in the social interaction domain were still common in most adult patients. In the communication and restricted repetitive behavior domains symptom abatement was more evident (Howlin et al. 2013). Functional outcome in terms of independent living, employment status and social relationships was restricted for most individuals with ASD (e.g. Howlin et al. 2013; Gray et al. 2014). Childhood IQ and comorbid medical disorders, more than autism symptom severity, were associated with later outcome (Howlin et al. 2013).

There is to our knowledge only one longitudinal study on milder forms of ASD (i.e., PDD-NOS) in a sample without intellectual disability (IQ above 70). Louwerse et al. (2015) compared ADOS scores in childhood (ages 6–12) and at follow up in adolescence (ages 12–20) in a group of 72 children with a clinical PDD-NOS diagnosis. Although ASD symptom severity showed a large within stability, an important percentage of the participants both in childhood (46%) and adolescence (43%) did not meet criteria when applying the dichotomous ADOS ASD classification. Special educational needs and mental health care use were high, and number of reciprocal friendships were low in both assessment waves, especially in adolescents with a higher level of ASD symptom severity. The study had no clinical reference group. We conclude that there is limited information on the adult outcome of ASD especially where this concerns individuals on the milder end of the spectrum (like PDD-NOS in DSM-IV), both on symptomatic and on functional aspects.

One aspect of information that may contribute to our understanding of adult ASD is the use of multi-informant data. Self-report data provide information on the subjective experience of autistic symptoms which are lacking in the above-mentioned studies. Other-report data potentially provide more information on behaviors that can be observed given limited insight of (some) persons with ASD in their social and repetitive behavior (Horwitz et al. 2016). In child psychiatry research on agreement and differences observed among multiple informants’ report of behavior has a long tradition, with consensus on the need to have both perspectives for a comprehensive view on the child’s problems. This is in contrast to research and clinical practice in adult psychiatry which mostly uses self-report. Parents tend to report more autistic behaviors in their underage children with ASD than these children report themselves (e.g. Johnson et al. 2009; Lerner et al. 2012). Multi-informant data in adults are scarce. Differences in self- and other-report of autistic behavior between adults with ASD and those with other psychopathology may increase our understanding of the differential diagnosis of this behavior in adults.

This also holds for comorbid psychopathology, in particular in relation to differentiation from other mental disorders. A growing body of evidence suggests a high rate of psychiatric comorbidity in children, adolescents and adults with ASD (Lai et al. 2019). Comorbidity is linked with symptom severity (e.g., Sprenger et al. 2013). Studying a group of children with PDD-NOS, De Bruin and colleagues (2007) concluded that compared to those without comorbid psychiatric disorders, children with a co-morbid disorder had more deficits in social communication. Comorbidity in childhood has been shown to influence the course of ASD symptomatology and to be associated with poorer functional outcome and quality of life (e.g. Mazzone et al. 2013). Four studies have systematically assessed the presence of comorbid psychiatric conditions in adults with ASD by semi-structured diagnostic interviews. A majority of the patients met criteria for more than one lifetime comorbid disorder, most notably major depressive disorder (54–77%), anxiety disorders (50–59%), and ADHD (30–69%). These rates were comparable to a clinically referred sample without ASD (Joshi et al. 2013). No differences in comorbidity rates were found between males and females (Hofvander et al. 2009), and between individuals diagnosed with ASD in childhood and those who were diagnosed with ASD in adulthood (Lugnegård et al. 2011). Lever and Geurts (2016) reported that ASD severity, as indicated by the Autism Spectrum Quotient (AQ: Baron-Cohen et al. 2001) self-report and ADOS score, was predominantly associated with anxiety severity and less with an anxiety diagnosis. Number of comorbid diagnoses did not seem to have a significant influence on daily functioning and subjective wellbeing (Lever and Geurts 2016).

The aim of this study was to investigate how patients who had received an earlier PDD-NOS or Asperger diagnosis before the age of 19 in specialist child and adolescent mental health care differed from other patients without ASD referred to specialist healthcare, with respect to current ASD symptoms (self- versus other-reported), comorbid diagnoses, and functional outcomes (mental health care use, medication use, educational attainment, social functioning). Data came from the clinical cohort of the Tracking Adolescents’ Individual Lives Survey (TRAILS CC; Oldehinkel et al. 2015). Respondents were originally recruited at age ~ 11 based on previous referral to a secondary care mental health outpatient clinic; we currently report on them at age ~ 19.

## Material and Methods

### Sample

#### TRAILS

The study is based on data from the clinical cohort of the Tracking Adolescents’ Individual Lives Survey (TRAILS CC), a prospective study aiming to explain the development of mental health from early adolescence (approximately age 11) into adulthood, with bi- or triennial assessments. The clinical cohort for TRAILS runs parallel to the TRAILS general population cohort. The TRAILS study has been described in detail elsewhere (Oldehinkel et al. 2015). The clinical cohort started in 2004 and consists of 543 individuals who had been referred to a child psychiatric outpatient clinic in the Northern Netherlands any time before the age of 11. The data we present here were mainly collected in the fourth assessment wave of TRAILS at which the participants were approximately 19 years old.

The TRAILS CC data were linked to the Psychiatric Case Register Northern Netherlands (PCRNN). The PCRNN registers specialist child, adolescent and adult mental health care consumption in the three Northern provinces of the Netherlands (population 1.7 million), which overlaps with the area from which TRAILS participants were recruited. Children and their parents gave consent to link their TRAILS data to health care records in the PCRNN. The PCRNN contained data from 2000 up (4 years preceding the start of TRAILS) on clinical diagnoses.

**Fig. 1:**
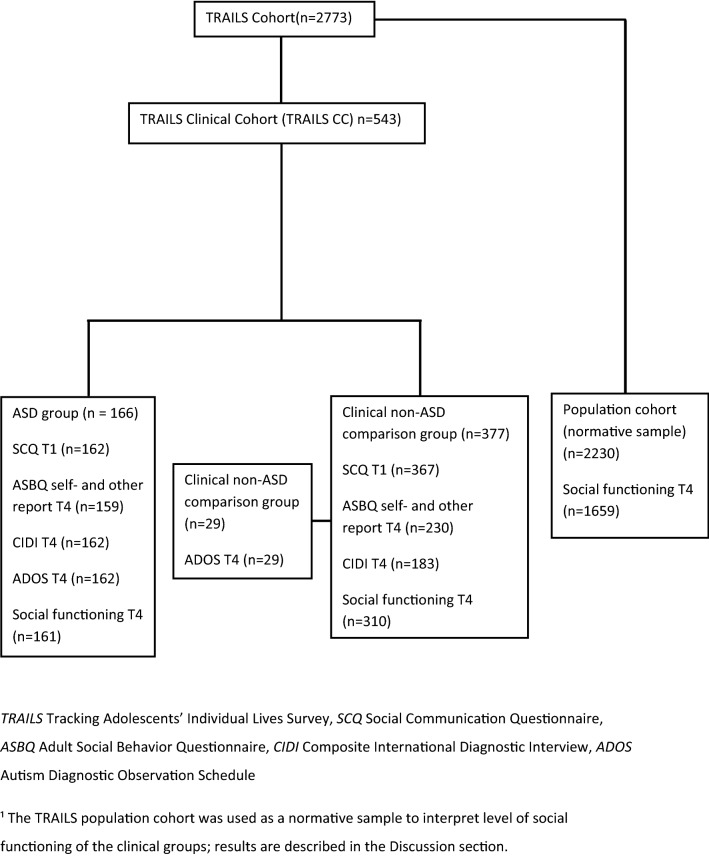
Flow chart showing how the study samples were selected from the TRAILS cohort

In the present study we compared 162 participants with an autism spectrum diagnosis (Asperger or PDD-NOS) diagnosed before the age of 19 to the remainder of the participants (n = 377) in the TRAILS CC cohort who were referred for different psychiatric problems (Fig. 1). At age 19, 389 participants and their parents completed self- and other rating version of the ASBQ, and 345 completed the Composite International Diagnostic Interview (CIDI, Kessler et al. 2004), all at the fourth assessment wave.

As an add-on study in TRAILS CC, we invited all participants with a DSM-IV classification 299.80 (Asperger's Disorder or Pervasive Developmental Disorder- not otherwise specified) according to the PCRNN (n = 162) to take part in the ADOS assessment. In addition, we selected a random comparison group of 29 participants from the non-ASD diagnosed part (according to the PCRNN) of the clinical cohort to compare ADOS scores. Most of them had a primary diagnosis of ADHD (n = 18), the other participants in this group had a wide variety of primary psychiatric diagnoses.

The current study also used data on the functional status of participants from the general population sample of TRAILS. This was done for educational attainment and number of social contacts, with the aim to compare findings to normative reference values from the general population.

### Measures

#### Intelligence, Demographic Data

The ASD and non-ASD clinical comparison group were compared on intelligence and demographic characteristics as measured at age 11 (baseline). Intelligence was measured on the basis of the Vocabulary and Block Design subtests of the Revised Wechsler Intelligence Scales for Children leading to approximate IQ scores (Silverstein 1967). Further, we measured the following socio-demographic variables at age 19: gender, age, parental SES, educational attainment, use of medication, consultation of mental health care in the last six months before assessment, current job or type of benefits, and number of social contacts. Self-report scores were supplemented with parent-report scores where appropriate.

#### ASD Questionnaires

The presence of ASD symptoms was assessed at baseline (age 11) using the *Social Communication Questionnaire* (SCQ; Berument et al. 1999) and at wave 4 (age 18–19) using the *Adult Social Behavior Questionnaire* (ASBQ; Horwitz et al. 2016). The SCQ is a screening tool to identify children at risk for ASD. Its 40 items are based on the Autism Diagnostic Interview—Revised (ADI-R, Lord et al. 1994) and focus on the presence of ASD behaviors during early childhood. The authors defined a cutoff value of 15 for ASD with a sensitivity of 0.85 and a specificity of 0.75. Schwenck and Freitag (2014) found an optimal cutoff value of 14–15 to differentiate ASD and ADHD in children with a normal intelligence (sensitivity 0.68, specificity 0.92), and an optimal cutoff of 11 to differentiate children with ASD and typically developing children in this group (sensitivity 0.92 and specificity 0.87). We used the SCQ scores to determine the likelihood of an ASD diagnosis, and its severity, based on developmental history.

The ASBQ is a quantitative measure of autistic traits with subscales that allow a differentiated description of ASD problems. Factor analysis provided support for six homogeneous subscales that concurred in the self- and other-report versions: *reduced contact, reduced empathy, reduced interpersonal insight, violation of social conventions, insistence on sameness* and *sensory stimulation/motor stereotypies*. Reliability estimates and correlations between self- and parent-ratings were good and the score profile on the 44-item ASBQ differentiated a group with ASD from a non-clinical group and patients with other diagnoses (Horwitz et al. 2016). We used both the self- and other-report version.

#### Observation of ASD Behavior

The *Autism Diagnostic Observation Schedule*—Module 4 (ADOS, Lord et al. 1999, module 4 for verbally fluent adults) is a standardized, semi-structured observational assessment of social interaction, communication and restricted and repetitive behaviors that is used in the diagnosis of ASD. Although the ADOS is not designed to be used in isolation in the diagnostic assessment of ASD, its discriminant validity in a population with a milder form of the spectrum has been reported as excellent (Fusar-Poli et al. 2017). An ADOS classification was originally based on thresholds on Social Interaction, Communication and on their combination (original algorithm, Lord et al. 1999). In 2014, Hus and Lord published a revised algorithm, containing a Social Communication domain and a Repetitive Restricted Behavior domain (revised algorithm, Hus and Lord 2014). They found satisfactory sensitivity of 90% and specificity of 82% of the classification, and confirmation of the applicability of the two-domain structure of the DSM5 in the revised algorithm of module 4. In the current study the ADOS was administered and scored by trained and certified psychologists.

#### Psychiatric (Co-)Morbidity

TRAILS assessed the presence of mental disorders based on the Diagnostic and Statistical Manual of Mental Disorders, fourth edition (DSM-IV, APA 2000) at T4 using the computer -assisted *CIDI 3.0*. The World Mental Health Organization Composite International Diagnostic Interview (CIDI, Kessler et al. 2004) is a structured diagnostic interview that can be administered by trained lay interviewers. The CIDI 3.0 assesses age of onset and 30-day, 12-month and lifetime prevalence estimates of any disorder. In this study we used the lifetime prevalence estimates. Based on Ormel et al. (2014) the diagnoses were grouped according to four major diagnostic classes: mood disorders (major depressive disorder, dysthymic disorder, and bipolar disorder I and II), anxiety disorders (panic disorder, agoraphobia, social phobia, specific phobia, generalized anxiety disorder, separation anxiety disorder, and obsessive–compulsive disorder), behavior disorders (attention deficit/hyperactivity disorder, oppositional defiant disorder, and conduct disorder) and substance use disorders (alcohol abuse/dependence, drug abuse/dependence). Eating disorders, schizophrenia, personality disorders, adjustment disorders and autism spectrum disorders were not assessed in the TRAILS CIDI.

### Analyses

Descriptive analyses were conducted to examine the frequency of outcomes across the diagnostic subgroups. Differences between the ASD group and the clinical non-ASD comparison group means on demographic variables were examined using Student's *t* test or chi-squared test, as appropriate. Group differences in SCQ scores and ASBQ self- and other- report scores between the ASD group and the clinical non-ASD comparison group were analyzed using multivariate general linear modeling (GLM) on the subscales of the SCQ and the ASBQ simultaneously. This multivariate test was followed up by univariate GLM analyses per subscale of the SCQ and the ASBQ. We further examined the difference in ASBQ scores in participants with and without psychiatric comorbidity (‘any disorder lifetime’ on CIDI) in the ASD group by a *t* test.

Mean ADOS scores on the original and the revised algorithm of the ASD group were compared to those of a clinical non-ASD comparison group using Student *t* test. Subjects within the ASD group with a revised algorithm ADOS score above and under autism spectrum cut-off were compared on SCQ scores at T1 and comorbid psychiatric diagnoses according to CIDI lifetime scores at T4.

We further examined differences between ASD- and clinical non-ASD comparison groups in CIDI scores using Student *t* tests and functional status at T4 using chi-squared test.

Effect sizes of between group differences were calculated using Cohen's d, where d ≥ 0. 2 is considered ‘small’, d ≥ 0.5 is considered ‘medium’ and d ≥ 0.8 is considered ‘large’ (Rea and Parker 1992). We used an alpha level of 0.01 in the analyses.

## Results

Table 1 shows differences between the ASD and the clinical non-ASD comparison group in demographic characteristics and developmental history ASD status, and current health care use. A larger percentage of males comprised the ASD group than the clinical reference group. Higher IQ scores were found in the ASD group. Differences between the ASD group and the clinical non-ASD comparison group in SCQ scores were identified using a multivariate GLM analysis with group as a fixed factor and the three SCQ subscale scores as the dependent measures (F = 8.806; df = 3; p < 0.001). Subsequent univariate GLM analyses for the total SCQ, and the SCQ subscales separately, showed that groups differed from each other on the total SCQ scale (F = 30.227; df = 1; p < 0.001) and the subscales (all three univariate p values ≤ 0.001).

**Table 1:** Differences between the ASD and the non-ASD clinical comparison group on demographic and developmental history characteristics

	ASD group (n = 162)	Clinical non-ASD comparison group (n = 377)	Group differences (*t *test or chi-square test)	Effect sizes (Cohen's *d*)
Demographic characteristics				
Gender (% male)	74%	62%	*p* < 0.01	0.32
Age (years) T4 [mean (sd)]	19.06 (.70)	19.15 (.75)	n.s	− 0.12
SES T1 Lowest 25%	18%	27%	n.s	0.25
Middle 50%	52%	50%	n.s	0.04
Highest 25%	30%	23%	n.s	0.21
*IQ (WISC)* T1 [mean (sd)]	100.07 (14.65)	95.25 (15.67)	*p* = 0.001	0.32
Developmental history ASD status (age 11)				
SCQ social interaction [mean (sd)]	4.64 (3.52)	2.94 (2.86)	*p* < 0.001	0.53
SCQ communication [mean (sd)]	4.51 (2.36)	3.83 (2.14)	*p* = 0.001	0.30
SCQ stereotypic behavior [mean (sd)]	2.39 (2.00)	1.66 (1.82)	*p* < 0.001	0.38
SCQ total [mean (sd)]	12.29 (6.56)	8.92 (5.67)	*p* < 0.001	0.55

*PCRNN* Psychiatric Case Register North Netherlands, *WISC* Wechsler Intelligence Scale for Children, *SES* Social Economic Status, *sd* standard deviation, *n.s.* not significant

**Fig. 2:**
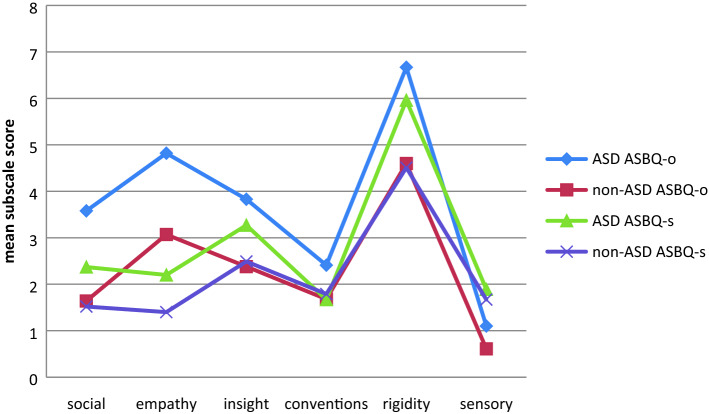
Mean ASBQ self- and other- report scores in ASD and non-ASD subjects

Table 2 and Fig. 2 show the mean ASBQ total and subscale scores per sample. A multivariate GLM analysis with group as a fixed factor and the six ASBQ subscales as the dependent measures showed an overall difference for both the other-report version (F = 7.872; df = 6; p < 0.001) and the self-report version (F = 3.509; df = 6; p = 0.002). Univariate GLM analyses for the total ASBQ, and the ASBQ subscales separately, indicated that groups differed from each other on parent scores on the total ASBQ scale (F = 31.717; df = 1; p < 0.001) as well as on all subscales (all six univariate p values < 0.001). For self report scores the differences were significant for the total ASBQ scale (F = 9.032; df = 1; p = 0.003) and the reduced contact scale (p = 0.001), the reduced empathy scale (p = 0.001) and the insistence on sameness scale (p = 0.007), but not for the reduced social insight scale, the violation of social conventions and the sensory stimulation & motor stereotypies scales*.*

**Table 2:** Differences between the ASD and the non-ASD clinical comparison group on other- and self report autistiform behavior [ASBQ; mean (sd)]

	ASD group (n = 159)	Clinical non-ASD comparison group (n = 230)	Group differences *F*^a^*p* values	Effect sizes (Cohen’s *d*)
Other report				
ASBQ contact	3.54 (3.25)	1.64 (2.39)	42.821	0.67
			*p* < 0.001	
ASBQ empathy	4.81 (4.13)	3.07 (3.78)	15.735	0.44
			*p* < 0.001	
ASBQ conventions	2.42 (2.97)	1.68 (2.43)	7.994	0.28
			*p* < 0.001	
ASBQ insight	3.87 (3.32)	2.38 (2.64)	21.864	0.50
			*p* < 0.001	
ASBQ rigidity	6.72 (4.44)	4.60 (4.31)	7.407	0.47
			*p* < 0.001	
ASBQ sensory	1.06 (1.91)	.60 (1.39)	21.445	0.26
			*p* < 0.001	
ASBQ total score	22.42 (15.21)	13.98 (13.23)	31.717	0.59
			*p* < 0.001	
Self report				
ASBQ contact	2.32 (2.64)	1.52 (1.95)	10.832	0.35
			*p* = 0.001	
ASBQ empathy	2.18 (2.55)	1.35 (2.10)	12.319	0.36
			*p* = 0.001	
ASBQ conventions	1.67 (1.82)	1.79 (1.76)	0.285	-0.06
			n.s	
ASBQ insight	3.30 (3.38)	2.49 (2.58)	5.992	0.27
			*p* = 0.015	
ASBQ rigidity	6.04 (4.36)	4.51 (3.78)	0.720	0.38
			*p* = 0.007	
ASBQ sensory	1.91 (2.58)	1.67 (2.30)	7.344	0.09
			n.s	
ASBQ total score	17.42 (12.60)	13.35 (10.60)	9.032	0.35
			*p* = 0.003	

*ASD* Autism Spectrum Disorder, *ASBQ* Adult Social Behavior Questionnaire, *contact* reduced contact, *empathy* reduced empathy, *conventions* violation of social conventions, *insight* reduced social insight, *rigidity* insistence on sameness, *sensory* sensory stimulation & motor stereotypies, sd standard deviation, n*.s.* non significant

^a^*df* = 1

Only thirty of the 162 patients in the ASD group exceeded ADOS ASD cut-off on the 'original algorithm', compared to 1 patient in the non-ASD subsample of TRAILS CC (n = 29). Using the new algorithm this ratio was 31 of 162 patients (the same 30 as with the original algorithm plus 1) in the ASD group and 1 of 29 (the same as with the original algorithm) patients in the control group. ADOS-2 scores on subscales and total scores according to both the original and the revised algorithm are shown in Table 3. Scores on all scales were higher in the ASD group, but differences on the communication scale (original algorithm) and the restricted repetitive behavior scale (revised algorithm) did not reach statistical significance.

**Table 3:** Differences between the ASD and the non-ASD clinical comparison group in scores on the Autism Diagnostic Observation Scale [ADOS-2, module 4; mean (sd)]

	ASD group (n = 162)	Clinical non-ASD control group (n = 29)	Group differences (*t* test)	Effect sizes (Cohen’s *d)*
Original algorithm				
Communication	1.43 (1.38)	1.10 (1.16)	n.s	0.26
Reciprocal social interaction	2.80 (2.34)	1.90 (1.45)	*p* < .001	0.46
Communication and social interaction (total score)	4.23 (3.17)	3.00 (2.08)	*p* < 0.01	0.39
Revised algorithm				
Social affect	3.21 (3.13)	2.03 (2.04)	*p* < 0.01	0.45
Restricted repetitive behavior	1.41 (1.47)	1.17 (1.29)	n.s	0.19
Social affect and restricted repetitive behavior (total score)	4.62 (4.05)	3.20 (2.44)	*p* < 0.01	0.38

*ASD* Autism Spectrum Disorder, *sd* standard deviation, *n.s.* not significant

A substantial lifetime prevalence of psychiatric comorbidity according to the CIDI (Table 4) was found in both groups and group differences were not significant. In the ASD group, ASBQ scores were on average higher in participants with a comorbid disorder (any DSM IV disorder according to the CIDI). These differences reached significance (p < 0.01; Student *t* test) in self-report on the subscales *violation of social conventions* (mean score in ASD participants with comorbidity 0.32 vs. 0.20 in ASD participants without comorbidity), *sensory stimulation & motor stereotypies* (0.28 vs. 0.16)*, insistence on sameness* (0.84 vs. 0.58) and the *total scale (*19.47 vs. 13.69)*.* Patients in the ASD group who exceeded ADOS ASD cut-off on the revised algorithm had fewer comorbid disorders according to the CIDI than those who scored under ADOS cut-off (Table 5).

**Table 4:** Differences between the ASD and the non-ASD clinical comparison group in the prevalence of lifetime psychiatric conditions (CIDI)

	ASD group (n = 162)	Clinical non-ASD comparison group (n = 183)	Group differences (chi-square test)	Effect sizes (Cohen’s *d*)
CIDI mood lifetime	27%	21%	n.s	0.14
CIDI anxiety lifetime	31%	41%	n.s	0.02
CIDI behavior lifetime	28%	24%	n.s	0.11
CIDIsubstance lifetime	15%	9%	n.s	0.07
CIDI any disorder llifetime	30%	56%	n.s	0.16

*ASD* Autism Spectrum Disorder, *CIDI* Composite International Diagnostic Interview, *mood* any mood disorder *anxiety* any anxiety disorder *behavior* any behavior disorder *substance* any substance disorder, *n.s.* not significant

**Table 5:** SCQ scores at T1, CSBQ scores at T1 and CIDI scores at T4 [mean (sd)] in ASD- ADOS positive and -ADOS negative subgroups

	ADOS above cutoff (n = 29)	ADOS under cutoff (n = 133)	Group differences (*t* test, chi-square test)	Effect sizes (Cohen’s *d*)
SCQ Total (T1)	13.82 (7.18)	11.17 (6.27)	n.s	0.39
CSBQ Total (T1)	33.34 (14.84)	32.14 (16.24)	n.s	0.08
CIDI lifetime T4				
0	57%	32%	*p* < 0.01	0.57
1	39%	31%	n.s	0.19
> 1	4%	37%	*p* < 0.01	-1.46

*SCQ* Social Communication Questionnaire, *CSBQ* Children’s Social Behavior Questionnaire, *ADOS* Autism Diagnostic Observation Scale, *CIDI* Composite International Diagnostic Interview, *sd* standard deviation, *n.s.* not significant

The far majority of participants in both groups participated in education, training or work, with only a few receiving social security or disability benefits (Table 6). We additionally compared the educational attainment of our clinical groups with the TRAILS normative population sample (not included in Table 6), and found that participants from both the ASD group and the clinical control group were less often in higher education than participants from the normative sample (18% and 17% vs. 34%), while more clinical participants than those from the normative sample were in vocational education (52% and 54% vs. 40%); these differences were statistically significant at *p* < 0.01 (2-sided *t* test). Most participants mentioned contacts with friends on a regular basis, in fact more so in the ASD group (approximately equal to the normative group) than in the clinical non-ASD comparison group. However, more parents from participants in the clinical groups than in the normative group estimated their child to have fewer friends than her son or daughter would have liked, with no differences between the clinical groups. More participants in the ASD group than in the clinical non-ASD comparison group used psychoactive medication or had contacts with specialized mental health service over the last 6 months.

**Table 6:** Differences between the ASD and the non-ASD clinical comparison group in functional outcomes at T4 (work, education, social contacts, mental healthcare use)

	PCRNN ASD group (n = 161)	PCRNN non-ASD group (n = 310)	Group differences (chi-square test)	Effect sizes (Cohen’s *d*)
Working	12%	7%	n.s	0.33
Disability benefit	6%	5%	n.s	0.11
Social welfare	1%	1%	n.s	0
Pre-vocational education	2%	1%	n.s	0
Vocational education	52%	54%	n.s	0
Higher secondary education	8%	12%	n.s	− 0.25
Higher education	18%	17%	n.s	0.04
Number of sick days in last 3 months [mean, (sd)]	2.9 (3.0)	1.7 (2.1)	n.s	0.46
Social contacts				
Meeting friends at home ≥ once a week	71%	49%	*p* < 0.01	0.52
Meeting friends outside home ≥ once a week	27%	29%	n.s	-0.05
Going out ≥ once a week	38%	31%	n.s	0.17
Having fewer friends than son/daughter would like (parent score)	15%	24%	n.s	-0.32
Current mental healthcare use				
On psychiatric medication	33%	16%	*p* < 0.01	0.27
On non-psychiatric medication	10%	13%	n.s	0.06
Contacts mental healthcare last 6 months	33%	11%	*p* < 0.01	0.39

*PCRNN* Psychiatric Case Register North Netherlands, *ASD* ASD group, *non-ASD* non-ASD group, *Norm* Normative group, *n.s.* not significant, *sd* standard deviation

## Discussion

In this study we investigated the symptomatic and functional characteristics of young adults who had been diagnosed with PDD-NOS or Asperger’s Disorder before age 19 in regular secondary mental health care. We compared their outcomes with those in a group of young adults previously diagnosed in the same mental health institution with other forms of psychopathology. We found that the self- and other report scores of autistic behavior on the ASBQ were higher in the ASD group, but not on all subscales. Most patients in this group did not meet the ADOS criteria for an ASD diagnosis in young adulthood. In both groups a high prevalence of multi-type comorbid psychiatric disorders was found. Compared to normative functioning, functional outcomes in terms of educational attainment and social contacts in the ASD group were less restricted than reported in previous studies.

Most young adults in our ASD group had received a PCRNN DSM-IV classification 299.80, which corresponds to a clinical diagnosis of PDD-NOS or Asperger's Disorder. Clinical experience in the Netherlands (Greaves-Lord et al. 2013) is that the classification 299.80 has been commonly used for the diagnosis PDD-NOS. The mean total SCQ score in the ASD group was 12.30, under the cutoff of 15 for ASD as defined by Berument et al. in their original study (Berument et al. 1999), but above the cutoff of 11 found by Schwenck and Freitag (2014) to optimally differentiate ASD and normal controls. This supports the impression that our ASD group consisted mainly of children on the milder end of the autism spectrum [severity level 1 according to the DSM-5 (American Psychiatric Association 2013)] or in some instances even outside the spectrum.

Although ASBQ scores in the present ASD group were higher than in the clinical non-ASD comparison group, scores in both groups were lower compared to those in a previous study by our group (Horwitz et al. 2016). This is consistent with the samples studied. In the previous study we included patients who were recently referred to a mental health care center and diagnosed with ASD, with high levels of distress. The current sample had had contact with an outpatient clinic some time before age 11; participants were, therefore, not recruited on the basis of ASD nor on referral at the start of the study or at age 19. Like in our previous study, we found higher scores in the ASD group compared to other diagnostic groups on the ASBQ other-report, here with small to medium effect sizes. These differences were smaller on the ASBQ self-report, probably due to reduced disease insight in patients with ASD (Bishop et al. 2012). In line with this, scores on the other-report subscales in the ASD group were higher than scores on the self-report subscales.

Consistent with the core problems of ASD in adulthood, differences between groups were largest on *reduced contact*, *reduced empathy*, *reduced social insight* and *insistence on sameness* subscales. On subscales *violation of social conventions* and *sensory stimulation & motor stereotypies* the differences between ASD and other groups were smallest (Table 2; Fig. 2). These results confirm the findings from our previous study on the ASBQ (Horwitz et al. 2016), in which we compared ASBQ self- and other report scores of patients with ASD with comparison groups ADHD, schizophrenia and depression and a non-clinical group. The fact that in the clinical reference group ADHD was a common diagnosis may explain the limited difference on the *violation of social convention* scale. ASD and ADHD show an overlap at the behavioral level; they have shared struggles with social rules, in ADHD expressed as social rudeness due to impulsivity and disinhibition (Nijmeijer et al. 2008) rather than reduced understanding of social rules. Given that the revised criteria in the DSM-5 allow a combined ASD and ADHD classification and behaviors pertaining to these diagnoses might look similar, accurate analysis of the context and meaning of these behaviors to determine whether they are part of ASD, ADHD or both is necessary to adequately treat and coach patients. The use of information gathered from both from the patient and from informants (by means of psychiatric interviews and questionnaires) can improve the quality of the diagnostic process*.*

Considering the low scores on the *sensory stimulation & motor stereotypies* subscale, the behavior and experiences tapped by this scale are relatively uncommon in less severe forms of ASD. We conclude that young adults with a previous PDD-NOS or Asperger diagnosis differ from other diagnostic groups on the social and insistence on sameness problem domains, but not on sensory-motor stereotypies nor on overt out of context social acts.

We expected that in this sample with relatively mild autistic behavioral problems in childhood, few of the patients would receive an adult ADOS ASD classification (cf. Louwerse et al. 2015), but that there would be higher average scores in the ASD group compared to the comparison group on a dimensional ADOS score. We further expected the largest differences with the comparison group on the domain of social interaction and smaller differences on restricted and repetitive behavior as measured by the ADOS (Billstedt et al. 2007; Chowdhury 2010; Howlin et al. 2013). We found the scores on the ADOS to be higher in our ASD group when compared to our clinical non-ASD comparison group according to both the original and the revised algorithms, but overall considerably below the ASD cutoff on the SA and RBB scales. The difference in terms of the effect size was the smallest on the restricted repetitive behavior scale of the revised algorithm. Furthermore, 81% of our ASD group fell outside the spectrum. In recent years several studies have been published regarding differences in ADOS scores according to both algorithms between patients with ASD and clinical comparison groups (Hus and Lord 2014; Pugliese et al. 2015; de Bildt et al. 2016; Fusar-Poli et al. 2017). These studies showed a wide range of ASD clinical severity in the ASD groups. Comparing the revised algorithm scores reported in these studies with those currently found, the social affect scale of both the ASD and clinical non-ASD comparison groups in the previous studies were higher than in our ASD group, while the restricted repetitive behavior scores were similar to our results. Although we found no association between ADOS and ASBQ scores, in accordance with previous studies that did not find a relation between scores on an ASD questionnaire and observation scales (e.g. Ashwood et al. 2017; Morrier et al. 2017; Lever and Geurts 2018), this finding parallels the ASBQ finding that our ASD group differed from the clinical non-ASD comparison group particularly in the social domain. We conclude that our young adults with a previous clinical PDD-NOS or Asperger diagnosis experienced higher levels of ASD problems, observed in particular by their parents, but that few met the formal criteria of the ADOS. Livingston and Happé (2017) recently suggested that the ADOS assessment situation (structured one-to-one interaction in a quiet space) may provide an optimal environment for compensation of autistic social-cognitive difficulties. In more challenging social situations in real life, this compensation might fail, and behavioral problems can subsequently become manifest. This may at least in part explain the discrepancy between ADOS and ASBQ scores. Another part of the explanation is that the ASD questionnaires sample behaviors over longer periods of time, while the duration of observation in the ADOS is brief. Nonetheless, the current view is that to confirm or dismiss an ASD diagnosis the ADOS score should be part of a comprehensive clinical assessment (Kamp-Becker et al. 2018). Our findings suggest that the ADOS may identify relatively severe ASD problems and not the mild problems.

We expected that the ASD group would have substantial comorbid psychiatric diagnoses (Hofvander et al. 2009; Lugnegård et al. 2011; Joshi et al. 2013; Lever and Geurts 2016) and wanted to investigate potential differences from the clinical non-ASD comparison group. We found that the majority of both the participants with- and without ASD had at least one lifetime comorbid psychiatric disorder, and there were no differences in the prevalence of the various disorders assessed by the CIDI. This confirms findings in previous studies regarding comorbidity in adults with ASD and extends this for current milder forms, as has also been reported for children with PDD-NOS (Greaves-Lord et al. 2013). Mood and anxiety disorders occurred less in our group than in previous studies, but it is likely that part of the onsets of mood and anxiety disorders in our young adult group are still to come. The CIDI does not measure the presence of less prevalent psychopathology such as psychotic disorders and eating disorders. Because of the growing interest in the co-occurrence of ASD with these disorders in clinical practice it is an important subject for future research. Our results underline the conclusion of Louwerse et al. (2015) who emphasized the need to reevaluate psychiatric comorbidity during adolescence when a milder form of ASD is diagnosed in childhood.

Our results indicated that ASBQ scores in ASD participants with a comorbid disorder (any DSM IV disorder lifetime according to CIDI) were on average higher than in those without psychiatric comorbidity; these differences reached significance in self-report (not other-report) on the subscales *violation of social conventions, sensory stimulation & motor stereotypies, insistence on sameness*, and the *total score*. This finding might support the hypothesis that psychiatric comorbidity in ASD is related to more self-reflection and -insight (e.g. Huang et al. 2017). Alternatively, these higher scores may reflect the presence of more severe ASD problems in comorbid cases, as indicated by the self-reported ASD symptoms and the CIDI interview with the young adults themselves. That higher ASD scores go together with higher comorbid psychiatric problems has also been reported for children with PDD-NOS, based on parent report (Greaves-Lord et al. 2013; Verheij et al. 2015). However, our ASD participants with an ADOS score under ASD cut-off had more psychiatric disorders according to CIDI score at T4 than those with an ADOS score above ASD cut-off. This finding suggests that the psychopathological symptoms of these patients with a previous PDD-NOS or Asperger diagnosis later on in their life are better accounted for by another diagnosis. This is in contrast to those with persistent ASD according to ADOS who report less comorbidity.

Various studies have reported the functional outcome of ASD in adulthood to be poor or very poor (e.g. Smith et al 2012; Anderson et al. 2014), even though a considerable heterogeneity in course has been found. Compared to these findings, social and educational outcome at age 19 in our ASD group appears to be less restricted when compared to our clinical non-ASD comparison group and the normative group. Number of social contacts did not differ significantly between groups, nor did the number of participants with a disability benefit. On the other hand, the percentage of young adults who received higher secondary education in the normative TRAILS group was higher (34%) than in both clinical groups, in the latter more participants received vocational education. When taking into account that IQ in our ASD group was higher than in the non-ASD group, educational outcome may be considered to be less than optimal. Further, the number of participants who had contacts with mental healthcare and who were on psychiatric medication was significantly higher in the ASD group than in the clinical non-ASD comparison group suggesting a marked vulnerability to develop psychiatric symptomatology that warrants therapeutic interventions. Our results bear some resemblance to those reported by Fein et al. (2013) on “optimal outcome” (OO) In their study 34 normal intelligent participants with a documented ASD diagnosis before the age of 5 who had lost their diagnosis at an average age of 13 were then assessed by ADOS and other instruments. In adolescence no differences in social and language functioning and academic abilities were found when the OO group was compared to a group of typically developing individuals (Troyb et al. 2014), but OO participants showed subtle deficits in attention and self-control and less insight into social relationships (Orinstein et al. 2015a). Further, OO youth showed more past and current psychiatric symptoms and diagnoses than typically developing youth, especially attention and impulsivity symptoms (Orinstein et al. 2015b). The same applies to the study by Louwerse et al. (2015) in which they found stability of ADOS symptom severity from childhood into adolescence in a normal intelligent PDD-NOS group referred to a university center. A large part of this group (46%) did not meet ADOS criteria for ASD classification in adolescence, but mental health care use and special educational needs were high. We refer to two viewpoints described in the recent literature that may aid in the interpretation of our findings. Mandy (2017) suggested a moderating role of autistic traits in the development of non-autistic psychopathology, which is, for example, apparent when a lack of mentalizing abilities and empathy may lead to the escalation of Oppositional Defiant Disorder traits into later antisocial behavior. From a transdiagnostic perspective, Van Os and Guloksuz (2017) hypothesized, in their critique of the “ultra high risk paradigm” (according to which the presence of psychotic experiences in youth pose a risk a for transition to schizophrenia), that psychotic experiences can be present in varying degrees as an early expression of nonspecific, multidimensional psychopathology. Just as Van Os and Guloksuz consider psychotic experiences to be a marker for the severity of non-psychotic psychopathology, autistic behaviors in children and adolescents with normal intelligence may have a similar meaning. More longitudinal studies are needed that focus on the course of multiple dimensions of psychopathology through adolescence to clarify the relationship between autistic traits and other psychopathology. Our findings support the DSM-5 delineating more stringent criteria for the diagnosis of ASD. A recent study into the symptom level of autism in 9-year old twins in Sweden indicated that over a decade, fewer autism symptom were required for a clinical diagnosis of autism (Arvidsson et al. 2018). Part of the group (who received their diagnosis based on DSM-IV) might not have been diagnosed with ASD according to the current rules of DSM-5. A study in childhood on the status of PDD-NOS in relation to DSM-5 showed that subgroups of children with PDD-NOS would fit into categories of Social Communication Disorder, Disruptive Mood Dysregulation Disorder, and ADHD, respectively (Greaves-Lord et al. 2013).

An important limitation of this study is that initial ASD diagnosis was made clinically according to the PCRNN and assessment was not systematically confirmed by us at that point in time by standardized diagnostic measures. The quality of diagnostic information obtained from the Case Register depends on clinical diagnostic practice, which in the Netherlands accords with the national clinical guideline stating that a best-estimate clinical diagnosis should be based on integrated information from different sources such as anamnesis, hetero-anamnesis and observation (Kan et al. 2013). The around-to-above threshold-levels of SCQ scores for ASD at the time of inclusion in the study at age 11 appear to confirm the presence of significant autistic behavior in the ASD group. Nonetheless, in particular in light of the current threshold in DSM-5, ASD may have been diagnosed too often. A second limitation is the diagnostic heterogeneity of the clinical comparison group*.* A third limitation of this study is that the CIDI was used to assess comorbid psychopathology. While this is a well-validated structured interview for diagnosing common adult mental disorders, there is little experience with this instrument when administered in individuals diagnosed with ASD. The fact that the prevalences of the various comorbid disorders were similar to those reported in the recent literature could tentatively be seen as an is indication of validity. Last, compared to previous studies, in which aspects like daily living skills and adaptive skills were examined, we explored a limited set of social and academic outcome variables, which means that no firm conclusions can be drawn about functional outcome.

In summary, this study shows that cognitively able young adults who were previously diagnosed with milder forms of ASD in secondary mental health care have higher scores on self-report, other-report and observation measures for ASD than clinical comparison subjects, but only a minority exceeds ASD cutoff according to ADOS. Psychiatric comorbidity does not differ between the groups, nor do social and educational functional outcomes, but the individuals diagnosed with ASD are more dependent on mental health care and young adults in this group use more psychiatric medication. Our results add to the growing body of evidence indicating that part of cognitive able individuals with an ASD diagnosis in their youth no longer the behaviors that underscribe a clinical ASD diagnosis in adulthood, yet still exhibit subtle difficulties in social functioning and a vulnerability for a range of other psychiatric disorders.
